# Single-nucleus gene and gene set expression-based similarity network fusion identifies autism molecular subtypes

**DOI:** 10.1186/s12859-023-05278-0

**Published:** 2023-04-11

**Authors:** Junjie Zhang, Guoli Ji, Xilin Gao, Jinting Guan

**Affiliations:** 1 Department of Automation, Xiamen University, Xiamen, Fujian China; 2 National Institute for Data Science in Health and Medicine, Xiamen University, Xiamen, Fujian China; 3 Xiamen Humanity Hospital, Fujian Medical University, Xiamen, Fujian China

**Keywords:** Single-nucleus RNA-seq data, Gene set, Similarity network fusion, Autism, Molecular subtype

## Abstract

**Background:**

Autism spectrum disorder (ASD) is a complex neurodevelopmental disorder that is highly phenotypically and genetically heterogeneous. With the accumulation of biological sequencing data, more and more studies shift to molecular subtype-first approach, from identifying molecular subtypes based on genetic and molecular data to linking molecular subtypes with clinical manifestation, which can reduce heterogeneity before phenotypic profiling.

**Results:**

In this study, we perform similarity network fusion to integrate gene and gene set expression data of multiple human brain cell types for ASD molecular subtype identification. Then we apply subtype-specific differential gene and gene set expression analyses to study expression patterns specific to molecular subtypes in each cell type. To demonstrate the biological and practical significance, we analyze the molecular subtypes, investigate their correlation with ASD clinical phenotype, and construct ASD molecular subtype prediction models.

**Conclusions:**

The identified molecular subtype-specific gene and gene set expression may be used to differentiate ASD molecular subtypes, facilitating the diagnosis and treatment of ASD. Our method provides an analytical pipeline for the identification of molecular subtypes and even disease subtypes of complex disorders.

**Supplementary Information:**

The online version contains supplementary material available at 10.1186/s12859-023-05278-0.

## Background

Autism spectrum disorder (ASD) is a severe neurodevelopmental disorder that is characterized by deficits in social communication and the presence of repetitive and restricted patterns of behaviors and interests [1]. ASD has significant phenotypic and genetic heterogeneity. Even though ASD is highly heritable, the genetic etiology is complex and influenced by over 1000 risk genes [2]. Together with environmental factors, it is challenging to diagnose and define ASD subtypes.

In the last decades, researchers have attempted to define subtypes of ASD. Traditionally, researchers first identify different clinical phenotypes and then identify and compare the biomolecular factors that may explain the differences in disease manifestation. For example, Diagnostic and Statistical Manual for Mental Disorders, version 5 (DSM-5) [3] defines subtypes of ASD, including Autistic Disorder, Asperger’s Syndrome, Childhood Disintegrative Disorder, and Pervasive Developmental Disorder-Not Otherwise Specified. However, the rapidly advancing genetic technology today still has difficulty in identifying genetic differences between these clinically behaviorally-defined subtypes, resulting in a lack of specific treatment options for them [4].

With the accumulation of sequencing data, studies are shifting to a genetic and molecular data-first approach for subtype definition [5], known as molecular subtype-first approach, which focuses on exploring molecular subtypes first based on genetic and molecular data [6]. This approach identifies recurrent genetic variants or expression patterns, reducing heterogeneity before phenotypic profiling [7]. Until now, molecular subtype analyses have defined some meaningful ASD molecular subtypes. For instance, a recent study identified a convergent molecular subtype of ASD with shared dysregulation across both epigenome and transcriptome [8]. Some ASD molecular subtypes caused by recurrent de novo disruptive mutations, such as *CHD8* [9] and *DYRK1A* [10], have also been reported.

Recently widely used single-cell RNA sequencing (scRNA-seq) and single-nucleus RNA sequencing (snRNA-seq) technologies have the advantage of detecting heterogeneity between cells and distinguishing different cell types. In 2019, Velmeshev et al. published snRNA-seq data of human brains from ASD patients and healthy controls and analyzed cell type-specific gene dysregulation in ASD [11]. ASD is characterized by cell type heterogeneity [12, 13], thus utilizing gene expression from multiple different cell types of human brains provides an unprecedented opportunity to more accurately identify ASD molecular subtypes. In addition, complementary to individual gene-based analyses, gene set-based analytical methods can better reveal the related gene sets whose components show subtle but coordinated expression changes that may not be detected by the usual individual gene-based analyses [14–16]. Considering that the presence of ASD-associated gene sets may determine the manifestation of ASD in different cell types, integrating gene and gene set analyses to mine ASD snRNA-seq data can broaden horizons for ASD molecular subtype identification.

To this end, we utilize similarity network fusion (SNF) [17] to integrate gene and gene set expression data of multiple human brain cell types for ASD molecular subtype identification. Then molecular subtype-specific gene and gene set expression patterns are analyzed, and the molecular subtypes are related to clinical diagnostic data to explore ASD disease subtypes. Finally, we construct ASD molecular subtype prediction models to aid clinical diagnosis and treatment. Our method provides new insights into the underlying genetic causes of ASD and can be also applied to the identification of molecular subtypes of other diseases.

## Materials and methods

### Single-nucleus RNA-seq data

We collected single-nucleus RNA-seq data of ASD and controls [11] from the website of https://autism.cells.ucsc.edu. The matrix of raw counts includes 104,559 nuclei of 15 ASD patients and 16 control individuals, involving 41 post-mortem tissue samples from anterior cingulate cortex and prefrontal cortex. These nuclei are classified into 17 cell types, including fibrous astrocytes (AST-FB), protoplasmic astrocytes (AST-PP), endothelial, parvalbumin interneurons (IN-PV), somatostatin interneurons (IN-SST), SV2C interneurons (IN-SV2C), VIP interneurons (IN-VIP), layer 2/3 excitatory neurons (L2/3), layer 4 excitatory neurons (L4), layer 5/6 corticofugal projection neurons (L5/6), layer 5/6 cortico-cortical projection neurons (L5/6-CC), microglia, maturing neurons (Neu-mat), NRGN-expressing neurons I (Neu-NRGN-I), NRGN-expressing neurons II (Neu-NRGN-II), oligodendrocytes and oligodendrocyte precursor cells (OPC). We preprocessed the raw data with R package scran [18], normalized and log transformed the gene expression data. After excluding the mitochondrial and nuclear genes, 11,559 highly variable genes were kept. After excluding the cell types with low nucleus numbers and severe imbalance in nucleus numbers between ASD patients, the gene expression data of 15 cell types were analyzed for downstream analyses, including 3662, 7089, 2001, 3713, 4180, 1834, 5620, 12,809, 6517, 3405, 4395, 2502, 3543, 12,214, and 9652 nuclei from cell types of AST-FB, AST-PP, endothelial, IN-PV, IN-SST, IN-SV2C, IN-VIP, L2/3, L4, L5/6, L5/6-CC, microglia, Neu-mat, oligodendrocytes, and OPC, respectively.

### Gene set variation analysis

Gene set variation analysis (GSVA) [19] is a non-parametric and unsupervised method for assessing the enrichment of transcriptomic gene sets by calculating sample-wise gene set enrichment scores and estimating variation of gene set enrichment over the samples. It converts an expression matrix of genes into an expression matrix of gene sets to assess whether gene sets are differently enriched between samples. We chose three categories of annotated gene sets in molecular signatures database (MSigDB) [20], including hallmark gene sets (H), the commonly used pathway gene sets in curated gene sets (C2) including KEGG [21], REACTOME and BIOCARTA, and gene ontology gene sets (C5). For each cell type, we supplied the gene expression matrix as well as the selected gene sets to an R package of GSVA for scoring gene sets for each cell, i.e., obtaining gene set expression level of each cell. The parameter *kcdf* was set to Gaussian, and *min.sz* was set to 10.

### Similarity network fusion

Similarity network fusion (SNF) [17] is a comprehensive method commonly used to integrate different modal data to identify cancer subtypes. SNF constructs similarity networks of samples for each modal data and then fuses the networks into a final one. To classify ASD molecular subtypes, the R package SNFtool [22] was used to perform SNF analysis to integrate gene and gene set expression data of all cell types to obtain a patient-patient similarity matrix.

For the gene expression data of cells in each cell type, we first calculated the average expression of each patient across all cells. Then we used the *affinityMatrix* function to calculate a patient-patient affinity matrix for each cell type, setting the number of neighbors *K* = 3. Next, we used the *SNF* function to fuse affinity matrices of all cell types, setting the number of neighbors *K* = 3 and the number of iterations *T* = 100. Based on the fused affinity matrix, patients were clustered using spectral clustering. We used the *optimumNumberOfClustersGivenGraph* function in SNFtool, i.e., utilizing eigen-gaps [22], rotation cost [22], and also calculated silhouette coefficient [23] to determine the optimal number of clusters, i.e., the number of ASD molecular subtypes. For the gene set expression data, we used the same way to obtain a fused affinity matrix of all cell types and to determine the number of clusters. Then, a final patient-patient affinity matrix was generated using the *SNF* function again, integrating the affinity matrix based on the gene expression matrix and that based on the gene set expression matrix.

### ASD molecular subtype-specific analyses

Based on the identified ASD molecular subtypes, we analyzed subtype-specific differentially expressed genes (DEGs) and differentially expressed gene sets (DEGSs). For each molecular subtype and each cell type, we firstly identified the DEGs for cells from patients belonging to the considered subtype relative to cells from patients belonging to the other subtypes, and also identified the DEGs for cells from patients belonging to the considered subtype relative to cells from normal controls. The intersection of these two sets of DEGs was taken as the subtype-specific DEGs. For this, MAST [24] was used to perform zero-inflated regression analysis by fitting a linear mixed model, which included molecular subtype group, individual label, gene detection rate, age, sex, RIN (RNA integrity number), PMI (post-mortem interval), cortical region, as well as 10X capture and sequencing batches and per-cell ribosomal RNA fraction. To identify genes differentially expressed due to the influence of molecular subtype, a likelihood ratio test was performed by comparing the model with and without the designated molecular subtype factor. Genes with log2 fold change (logFC) of expression ≥ 0.14 (i.e., 10% difference) and FDR < 0.05 were regarded as differentially expressed. To identify the molecular subtype-specific DEGSs for each subtype and each cell type, we used R package limma [25] to analyze the gene set expression data obtained from GSVA. Gene sets with logFC of expression ≥ 0.14 and FDR < 0.05 were regarded as significant.

### Correlation analysis with clinical scores

We used the Autism Diagnostic Interview-Revised (ADI-R) data of patients obtained from the ASD snRNA-seq study [11], including scores of five categories: A, B-verbal, B-nonverbal, C and D, where A stands for social, B for communication, C for repetitive behavior and D for abnormal development. We ranked the scores of all ASD patients within each category and used the average of ranks of each patient as the combined clinical score. To analyze the correlation between subtype-specific DEGs/DEGSs and combined clinical score, for each gene of subtype-specific DEGs/DEGSs, we firstly calculated individual-level gene expression fold change using MAST by comparing each ASD case with the control group, and then calculated Pearson’s correlation coefficient and associated *P-*value between individual-level fold changes and combined clinical scores. Next, we determined the meta Pearson’s *P-*value by combining the *P-*values of all genes of subtype-specific DEGs/DEGSs using Fisher’s method [26]. Meta *P-*value was used as an approximation of how well the changes of genes correlate with the clinical severity of ASD. To analyze the different clinical ADI-R category in score ranks between one considered molecular subtype and the other subtypes, we performed *t* test for each category, i.e., A, B-verbal, B-nonverbal, C and D, and the *t* test *P*-values of all categories were corrected using Benjamini–Hochberg procedure [27] to obtain FDR-adjusted *P*-values.

### ASD molecular subtype prediction model

R package caret [28] was used to construct subtype prediction models for each cell type based on partial least squares (PLS) algorithm, using the genes of molecular subtypes-specific DEGs/DEGSs as features. We constructed three kinds of prediction models. For the first one, cells were randomly divided into a training set and a test set in a ratio of 7:3 for each molecular subtype. For the second one, cells from one randomly selected patient of each molecular subtype were used as a test set and cells from other patients as a training set to avoid information leakage. For these first two kinds of models, based on the split training set, we chose the optimal model by performing ten-fold cross-validation 10 times and tuning over the model hyperparameter, i.e. the number of PLS components, with a grid search. Then the optimal model was used for the prediction of the test set. For the third one, the gene expression average of all cells of one patient was used as the patient’s gene expression data and one randomly selected patient of each subtype was used as a test set and the left patients as a training set. Based on the split training set, we chose the optimal model by performing three-fold cross-validation 10 times and tuning over the model hyperparameter with a grid search. To evaluate the prediction performance, we calculated micro F1, macro F1, and weighted F1 as performance metrics. F1 score can be considered as a weighted average of the precision and recall of the model, taking values in the range of [0,1]. The larger the F1 score, the better the model prediction.

## Results

### Analytical workflow

**Fig. 1:**
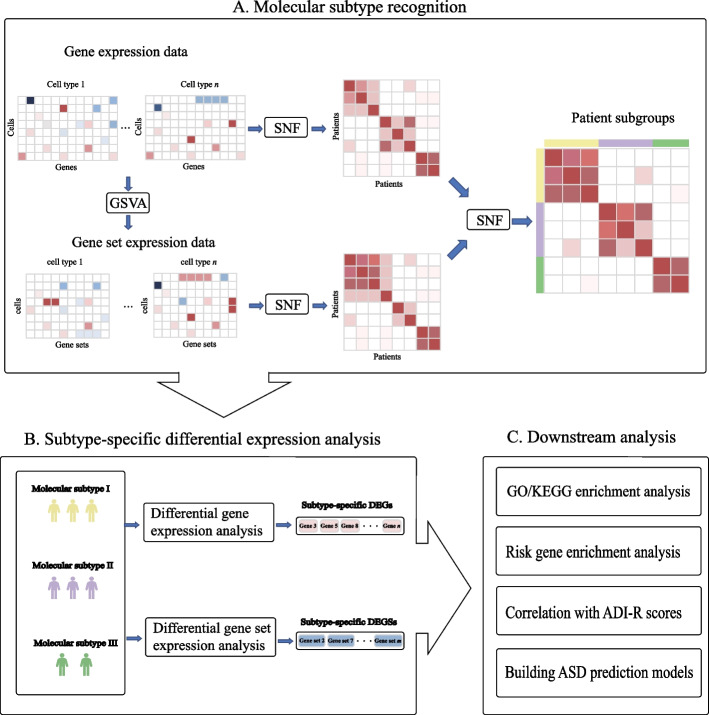
Analytical workflow including **A** molecular subtype recognition, **B** subtype-specific differential expression analysis, and **C** downstream analysis for subtype-specific DEGs and DEGSs

To integrate gene expression and gene set expression of multiple human brain cell types, we performed similarity network fusion (SNF) analysis. Specifically, based on the snRNA-seq data of ASD patients, SNF was applied to integrate the patient-patient similarity networks obtained from gene expression data of multiple cell types. At the same time, for each cell type, we supplied gene expression data and curated gene sets for gene set variation analysis (GSVA) to obtain gene set expression data, and then SNF was applied to integrate the patient-patient similarity networks obtained from gene set expression data of multiple cell types. Next, we used SNF again to integrate the above patient-patient similarity networks to get a final patient-patient affinity matrix. Then we performed clustering based on the affinity matrix to determine the molecular subtypes of ASD patients (Fig. 1A). Based on the identified molecular subtypes, we performed subtype-specific differential expression analyses including identifying differentially expressed genes (DEGs) and differentially expressed gene sets (DEGSs) for each cell type (Fig. 1B). We performed GO [29, 30] and KEGG [21] enrichment analyses of subtype-specific DEGs, ASD risk gene enrichment analysis and clinical score association analysis of subtype-specific DEGs and DEGSs. Finally, partial least squares (PLS)-based ASD molecular subtype prediction models were constructed (Fig. 1C).

### Identification of ASD molecular subtypes

**Fig. 2:**
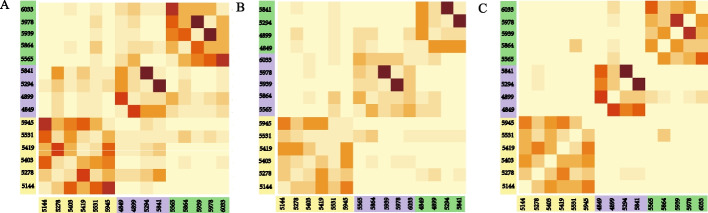
Patient-patient affinity matrix and clustering diagram. Patient-patient affinity matrices and clustering results based on **A** gene expression data and **B** gene set expression data. **C** The final patient-patient affinity matrix integrating (**A**) and (**B**) and the final clustering result

Using the gene expression data of multiple cell types, we used SNF to obtain a patient-patient affinity matrix. Based on eigen-gaps [22], rotation cost [22] and silhouette coefficient [23], the optimal number of clusters was set to three and three ASD patient clusters were determined (Fig. 2A). At this time, the silhouette coefficient was 0.63. Similarly, based on the gene set expression of multiple cell types, SNF was used to get a patient-patient affinity matrix. Three clusters were also obtained (Fig. 2B). The silhouette coefficient was 0.65. Afterward, we fused the above two affinity matrices by SNF to obtain a final patient-patient affinity matrix. Again, the optimal number of clusters was three and three ASD clusters were determined (Fig. 2C). At this time, the silhouette coefficient was 0.92. Noted that the clustering results are the same no matter in Fig. 2A, B, or C. Hence, we classified the 15 ASD patients into three molecular subtypes, denoted by ASD molecular subtype I, II, and III, including six, four, and five patients respectively.

### ASD molecular subtype-specific DEGs

**Fig. 3:**
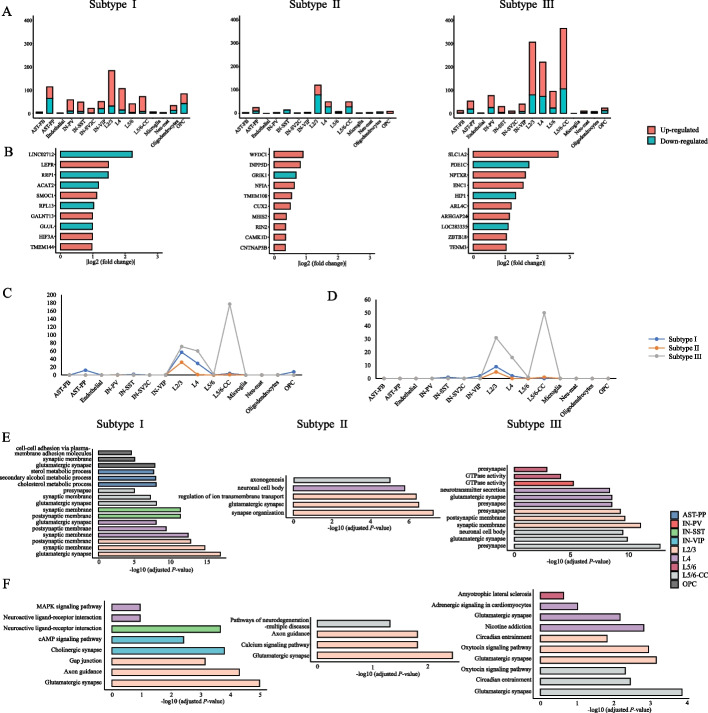
Analysis of subtype-specific DEGs. **A** The number of subtype-specific DEGs in each cell type. **B** The top 10 subtype-specific DEGs unique to each molecular subtype ranked by |logFC|. The regulation direction and |logFC| are relative to other molecular subtypes. The number of (**C**) GO terms [29, 30] and **D** KEGG pathways [21] enriched with subtype-specific DEGs in each cell type. The top three subtype-specific **E** GO terms [29, 30] and **F** KEGG pathways [21] ranked by adjusted *P*-value in each cell type

Based on the three recognized ASD molecular subtypes, we identified subtype-specific DEGs for each molecular subtype and each cell type (Additional file 1: Table S1). Figure 3A shows the number of up-regulated and down-regulated subtype-specific DEGs relative to other molecular subtypes in each cell type. The number of subtype-specific DEGs varies considerably among the three molecular subtypes, but all molecular subtypes have a large number of subtype-specific DEGs in L2/3, L4, and L5/6-CC. Subtype I has more subtype-specific DEGs in AST-PP and OPC, while subtype III has a large number of subtype-specific DEGs in L5/6-CC, implying the different influence of ASD on different cells for different molecular subtypes.

Then, we analyzed the subtype-specific DEGs unique to each molecular subtype (Additional file 2: Table S2), and listed the top 10 with the largest absolute value of logFC relative to other molecular subtypes (Fig. 3B). Previous studies have shown these subtype-specific DEGs are associated with ASD, for example, *LEPR* [31] and *GALNT1*3 [32] for molecular subtype I, *GRIK1* [33] and *CUX2* [34] for molecular subtype II, and *SLC1A2* [35] and *PDE1C* [36] for molecular subtype III. We also selected the subtype-specific DEGs possessed by all molecular subtypes and compared the logFC of these genes in corresponding cell types relative to controls to screen for genes with inconsistent regulatory direction among the three molecular subtypes. We detected 19 different regulatory orientation events in 17 unique genes (Additional file 2: Table S2). Among them, two ASD risk genes, *CNTN5* in subtype I and *CNKSR2* in subtype II, are of interest.

To explore the biological significance of the ASD molecular subtypes, we performed GO and KEGG enrichment analyses of subtype-specific DEGs using clusterProfiler [37] (Additional file 3: Table S3). After the correction of multiple tests using Benjamini–Hochberg procedure [27], GO terms with count > 10 and FDR-corrected *P*-value < 0.05 were reported. The number of significant GO terms in each cell type is shown in Fig. 3C. For the three subtypes, GO terms are enriched in six, three and five cell types, respectively. All molecular subtypes in L2/3 have a large number of enriched GO terms while only molecular subtype III also has many enriched GO terms in L5/6-CC. Listing the top three significant GO terms according to adjusted *P*-value in Fig. 3E, it shows that the majority of these GO terms are associated with neurological function. Similarly, KEGG pathways with count > 5 and FDR-corrected *P*-value < 0.05 were screened. Figure 3D shows the number of significantly enriched KEGG pathways. For the three subtypes, KEGG pathways are enriched in four, two and four cell types, respectively. All molecular subtypes also have a large number of enriched KEGG pathways in L2/3 while only molecular subtype III has many enriched KEGG pathways in L5/6-CC. The top three significant pathways are listed in Fig. 3F. Among them, most are related to neurological function. The results of GO and KEGG enrichment analyses show that the three molecular subtypes are closely related to ASD, while each has its characteristics.

### ASD molecular subtype-specific DEGSs

**Fig. 4:**
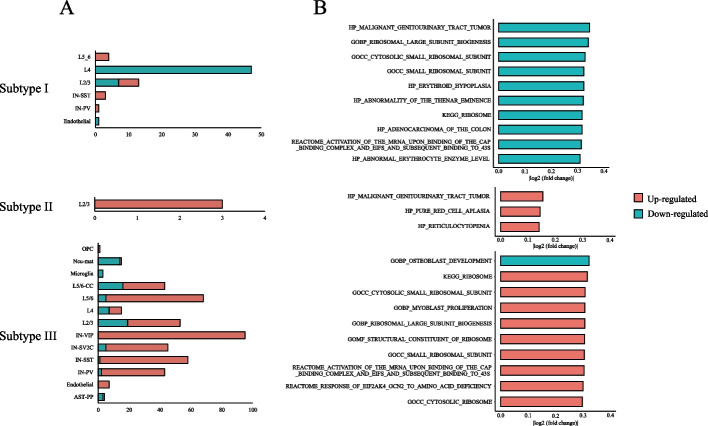
Analysis of subtype-specific DEGSs. **A** The number of subtype-specific DEGSs in different cell types. **B** The top 10 subtype-specific DEGSs ranked by |logFC| for the three molecular subtypes. The regulation direction and |logFC| are relative to other molecular subtypes

Based on the three identified ASD molecular subtypes, we also identified subtype-specific DEGSs for each molecular subtype and each cell type (Additional file 4: Table S4). As shown in Fig. 4A, the number of subtype-specific DEGSs varied greatly among molecular subtypes. Molecular subtype I has subtype-specific DEGSs in six cell types, with the most abundant in L4. Most of these subtype-specific DEGSs exhibits down-regulation relative to other molecular subtypes. Molecular subtype II has three subtype-specific DEGSs in L2/3 only that exhibits up-regulation relative to other molecular subtypes. Molecular subtype III has subtype-specific DEGSs in 13 cell types, with the most abundant in IN-VIP. Most of these subtype-specific DEGS exhibits up-regulation relative to other subtypes. The top 10 subtype-specific DEGSs with the largest absolute value of logFC relative to other molecular subtypes are shown in Fig. 4B. Among them, GOBP_RIBOSOMA_LARGE_SUBUNIT and GOCC_SMALL_ RIBOSOMAL_SUBUNIT in subtype I have been shown in previous studies to regulate biological functions that affect neurological development and may contribute to neurological disorders [38]. It also has been suggested that KEGG_RIBOSOME and GOBP_OSTEOBLAST_DEVELOPMENT in subtype III may influence the clinical phenotype of ASD [39].

Then, we detected the subtype-specific DEGSs unique to each molecular subtype (Additional file 5: Table S5). We also selected the common subtype-specific DEGSs across all molecular subtypes, a total of two gene sets, and compared the logFC of these gene sets relative to controls to screen for those with inconsistent regulatory direction among the three subtypes. Two different regulatory orientation events were detected (Additional file 5: Table S5). HP_PURE_RED_CELL_APLASIA and HP_MALIGNANT _GENITOURINARY_TRACT_TUMOR are down-regulated in molecular subtype I and up-regulated in the other subtypes relative to the control group. These gene sets may be used as marker gene sets to identify molecular subtypes.

### Association analysis between molecular subtypes and ASD

**Fig. 5:**
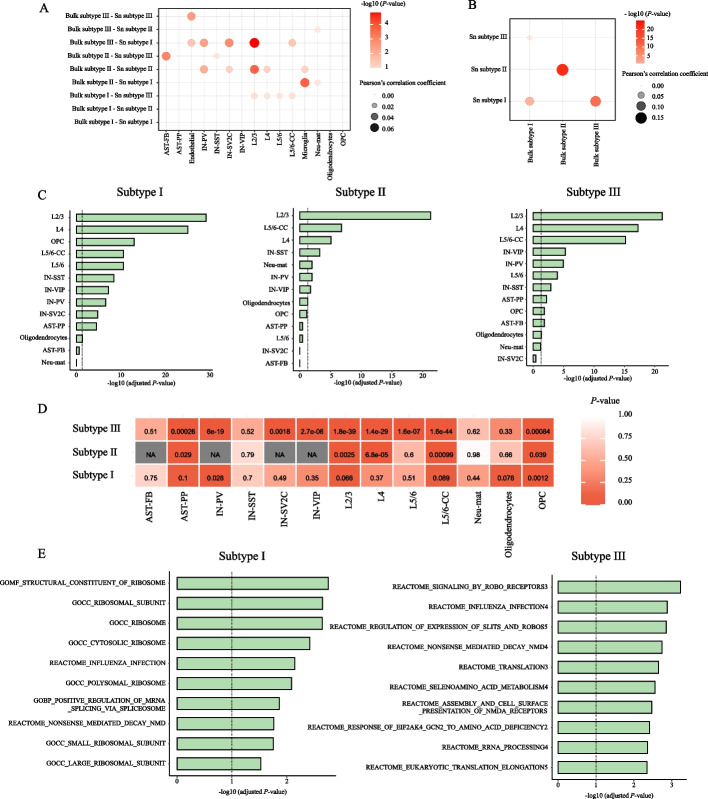
Association analysis between molecular subtypes and ASD. Pearson’s correlation coefficient and corresponding *P*-value between the fold changes of genes in each bulk subtype relative to other bulk subtypes and those in each Sn subtype relative to other Sn subtypes, calculated based on (**A**) single-nucleus gene expression data and **B** the combined and averaged single-nucleus data across all cell types by individual. **C** The degree of overlap between SFARI ASD genes and subtype-specific DEGs for the three molecular subtypes. The dotted line indicates the threshold of statistical significance, FDR adjusted *P*-value = 0.05. **D** Meta *P*-values of Pearson’s correlation between subtype-specific DEGs and ADI-R clinical scores in each cell type. NA indicates that the cell types were excluded because there are fewer than five subtype-specific DEGs. **E** The top 10 subtype-specific DEGSs having the most significant correlation with ADI-R scores ranked by -log10 (meta *P*-value). The dotted line indicates the threshold of statistical significance, meta *P*-value = 0.1

To validate the three identified molecular subtypes based on single-nucleus RNA-seq data (in this section we denoted as Sn subtypes), we used the human brain bulk RNA-seq data of 47 ASD samples (32 ASD individuals) and 57 controls (40 control individuals) [40] to identify ASD molecular subtypes (in this section we denoted as bulk subtypes) and then checked if the identified Sn subtypes could correspond to bulk subtypes. Specifically, after preprocessing the bulk gene expression, we supplied it to GSVA for generating gene set expression data, and then the gene and gene set expression data were fused with SNF to obtain a patient-patient similarity matrix. Based on the similarity matrix, three bulk subtypes were obtained, denoted as bulk subtypes I, II, and III. We calculated the Pearson’s correlation coefficient between the fold changes of genes in each Sn subtype relative to other Sn subtypes and those in each bulk subtype relative to other bulk subtypes. When considering the positive correlations, we found that in many cell types bulk subtype I is significantly positively correlated with Sn subtype III, bulk subtype II is more positively correlated with Sn subtype II, and bulk subtype III is positively correlated with Sn subtype I (Fig. 5A). Moreover, to mask the effect of cell types, we combined and averaged the single-nucleus data across all cell types by individual for each identified Sn subtype and then calculated the fold change of each gene in each Sn subtype relative to other Sn subtypes based on the combined data. By calculating the Pearson’s correlation coefficient between the fold changes of genes in each Sn subtype relative to other Sn subtypes and those in each bulk subtype, we also noted that Sn subtype I is more positively correlated with bulk subtype III, Sn subtype II is significantly positively correlated with bulk subtype II, and Sn subtype III is positively correlated with bulk subtype I (Fig. 5B). It can be seen that our identified molecular subtypes based on single-nucleus RNA-seq data could be verified by bulk RNA-seq data.

To further explore the extent to which the identified ASD Sn molecular subtypes are associated with ASD, we assessed the enrichment of curated ASD risk genes with molecular subtype-specific DEGs using hypergeometric tests. Two cell types with less than 10 subtype-specific DEGs, endothelial and microglia, were excluded. The ASD risk genes were obtained from Simons Foundation Autism Research Initiative (SFARI) (released on 22 July 2022), of which 429 genes are in our gene expression matrix. The molecular subtype-specific DEGs are significantly enriched with ASD genes (FDR adjusted *P*-value < 0.05) in the vast majority of cells (Fig. 5C, and Additional file 1: Table S1). For all three subtypes, ASD risk genes are the most overrepresented in L2/3. Moreover, we estimated the significance of the overlap between each molecular subtype-specific DEGS and SFARI ASD genes (Additional file 4: Table S4). HP_TYPICAL_ABSENCE_SEIZUR and REACTOME_NEUREXINS_AND_ NEUROLIGINS in molecular subtype III have the highest degree of overlap with ASD genes. The high degree of overlap between subtype-specific DEGs/DEGSs and ASD risk genes suggests a high correlation between the identified Sn molecular subtypes and ASD.

Next, to explore the associations between Sn molecular subtypes and clinical symptoms, for each gene of subtype-specific DEGs, we calculated Pearson’s correlation coefficient and corresponding *P*-value between patient-level fold changes relative to controls and combined ADI-R clinical scores of patients, and then obtained meta *P*-value to represent how well the changes of DEGs in a given molecular subtype correlate with clinical severity. We excluded two cell types with less than 10 subtype-specific DEGs, endothelial and microglia, and for molecular subtype II, we excluded the cell types with fewer than five subtype-specific DEGs. In many cell types, the subtype-specific DEGs correlate with the clinical severity (meta *P*-value < 0.1), indicating that the identified Sn molecular subtypes are associated with clinical symptoms (Fig. 5D). This suggests that in most cells, the clinical scores of the three subtypes are consistent with the underlying biomolecular mechanisms. Besides, we also performed an association analysis between each molecular subtype-specific DEGS and ADI-R clinical score. Molecular subtype I has 34 gene sets and molecular subtype III has 114 gene sets showing a high correlation with clinical scores. The top 10 significant subtype-specific DEGSs for subtypes I and III are shown in Fig. 5E. GOMF_STRUCTURAL_CONSTITUENT_OF_RIBOSOM of subtype I, and REACTOME_SIGNALING_BY_ROBO_RECEPTORS of subtype III have the highest correlation with ASD clinical scores.

As to the analysis of different clinical ADI-R category in score ranks between one considered Sn molecular subtype and the other subtypes, we performed *t* test for each category of ADI-R score and the *P*-values of all categories were corrected using Benjamini–Hochberg procedure [27] (Additional file 6: Table S6). It was noted that C (repetitive behavior) score rank of Sn molecular subtype I is lower than those of the other two molecular subtypes (*P*-value = 0.016, FDR adjusted *P*-value = 0.078). There may be also a tendency that A (social) score rank of Sn molecular subtype II is lower than those of the other subtypes (*P*-value = 0.069), though it is not significant. These observations may be helpful in the diagnosis of molecular subtypes of ASD, which then facilitates the treatment of ASD. The association analysis with ADI-R or other clinical data, linking ASD molecular subtypes with clinical manifestation, would be more practical when more clinical data become available in the future.

### ASD molecular subtype prediction

**Fig. 6:**
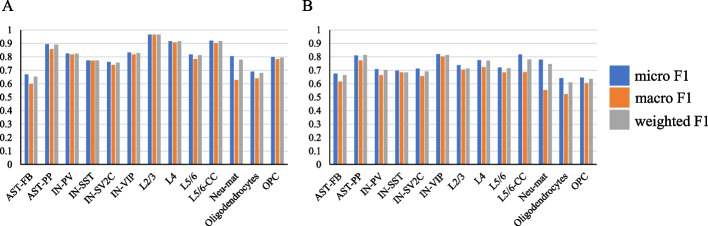
Performance metrics on test set. Performances of predictive models constructed using **A** randomly selected cells as the training set and test set, and **B** cells from one randomly selected patient as the test set and cells from other patients as the training set for each molecular subtype

Constructing machine learning-based disease prediction model can facilitate the clinical diagnosis of diseases [41–43]. Using the identified ASD molecular subtype-specific DEGs, we built PLS-based prediction models for classifying molecular subtypes. The data from endothelial and microglia were not used as there are few subtype-specific DEGs. For each cell type, the subtype-specific DEGs of all three subtypes were used as features to construct prediction models at the cell level based on cell gene expression for predicting the patient subtype a cell belongs to. Firstly, for each molecular subtype, cells were randomly divided into a training set and a test set in a ratio of 7:3. It can be seen that the performance is good in most cell types (Fig. 6A). Especially in L2/3, L4 and L5/6-CC, which have a high correlation with ASD, macro F1, micro F1 and weighted F1 on the test set can reach 0.9, proving that molecular subtype-specific DEGs have a good prediction. Secondly, for each molecular subtype, considering using cells from one randomly selected patient as a test set and cells from other patients as a training set, we constructed prediction models at the cell level again. The performances in AST-PP, IN-VIP and L5/6-CC are better than other cell types (Fig. 6B). This kind of data partitioning ensured that no information leakage occurs between training and test sets and the constructed model is of high practical value.

In addition, we also considered to constructed predictive models at the patient level. The gene expression average of all cells of one patient was used as the patient’s gene expression data. For each subtype, we randomly selected one patient as a test set and the left patients as a training set. Because the sample size is small and to avoid the effect of randomness, we repeated the data set partition and model construction ten times and then calculated the average prediction accuracy to assess the effectiveness. The average prediction accuracy values are 0.6 for IN-PV, and above 0.7 for all other cell types. This reflects that these three subtypes can be well distinguished even in a cell type with a small sample size.

As to molecular subtype-specific DEGSs, we also used them to build ASD subtype prediction models. Because only in L2/3 all three molecular subtypes possess subtype-specific DEGSs, we built prediction model only in L2/3 using the genes contained in all subtype-specific DEGSs as features. First, when 70% of cells from all patients were selected as the training set and the other cells were used as the test set, macro F1, micro F1 and weighted F1 are 0.72, 0.69 and 0.71, respectively. Second, when all cells from one patient in each molecular subtype were selected as the test set and cells from other patients were used as the training set, macro F1, micro F1 and weighted F1 are 0.81, 0.79 and 0.80, respectively. Finally, when one patient from each molecular subtype was selected as the test set and the other patients as the training set, the average accuracy of 10 training/testing replicates is 0.73. When more patient sequencing data become available, the construction of predictive models at the patient level to predict the molecular subtype of a patient will hopefully be realized.

## Discussion

The identification of ASD subtypes remains an unresolved challenge, which adds a barrier to the treatment of patients with ASD. Traditional methods of identifying ASD subtypes defined by clinical behaviors directly may just provide limited assistance in the genetic genesis and treatment of ASD. With the recent accumulation of biological sequencing data, studies are shifting to a genetic and molecular data-first approach to subtype definition, i.e., identifying molecular subtypes first. Considering the fact that ASD is characterized by cell type heterogeneity and the presence of ASD-associated gene sets may determine the manifestation of ASD in different cell types, our study innovatively integrated gene and gene set expression data of multiple human brain cell types using SNF to define ASD molecular subtypes.

To explore the characteristics of the identified molecular subtypes, we analyzed their cell type-specific and subtype-specific DEGs. All three subtypes have a large number of subtype-specific DEGs in L2/3, L4 and L5/6-CC, which is similar to the result of the ASD snRNA-seq study [11], indicating these cell types are mostly affected by ASD. Subtype I has more DEGs in AST-PP and OPC than the other two subtypes, while subtype III has more DEGs in L5/6-CC, implying the different influence of ASD on cell types for different molecular subtypes. The enriched GO terms and KEGG pathways with the subtype-specific DEGs are mostly related to neurological functions. Besides, subtype-specific DEGs for all molecular subtypes overlap significantly with ASD risk genes and show a high correlation with clinical symptom severity.

Except for subtype-specific DEGs, we also identified molecular subtype-specific DEGSs. The subtype-specific DEGSs of the three molecular subtypes are distinctive no matter in the number of identified DEGSs or in the regulatory orientation, with molecular subtype I having more down-regulated DEGSs in L4 and molecular subtype III having more up-regulated subtype-specific DEGSs in IN-VIP. Furthermore, some gene sets of molecular subtypes I and III show a high correlation with the clinical severity, such as GOMF_STRUCTURAL_CONSTITUENT_OF_RIBOSOM and GOCC_RIBOSOMAL_SUBUNI of subtype I.

The subtype-specific DEGs/DEGSs unique to molecular subtypes and the common subtype-specific DEGs/DEGSs across all subtypes but with different regulatory orientations can be used as distinguishing genes/gene sets for different molecular subtypes. To demonstrate their prediction ability, we constructed prediction models. Also, using the differences in clinical scores of the identified molecular subtypes would be helpful to diagnose the molecular subtype of ASD. Our results may aid in the identification and diagnosis of ASD molecular subtypes, and even disease subtypes. Our method can be further practically applied in the future when more ASD scRNA-seq/snRNA-seq data and clinical data are available.

## Conclusions

In this study, we identified ASD molecular subtypes by performing similarity network fusion to integrate gene and gene set expression data of multiple human brain cell types. Then we applied subtype-specific differential gene and gene set expression analyses to study expression patterns of different molecular subtypes. The identified molecular subtype-specific genes and gene sets may be used as biomarkers to classify ASD molecular subtypes, facilitating the diagnosis and treatment of ASD. Our method can also be applied for the identification of molecular subtypes and even disease subtypes of other complex disorders.

## Supplementary Information

**Additional file 1. Table S1:** Molecular subtype-specific DEGs information, the enrichment with SFARI ASD genes, and the association with clinical scores.

**Additional file 2. Table S2:** Molecular subtype-specific DEGs unique to subtypes along with the regulation direction and |logFC| relative to other molecular subtypes, and those differentially regulated among different molecular subtypes relative to controls.

**Additional file 3. Table S3:** GO and KEGG enrichment analysis of molecular subtype-specific DEGs.

**Additional file 4. Table S4:** Molecular subtype-specific DEGSs, the enrichment with SFARI ASD genes, and the association with clinical scores.

**Additional file 5. Table S5:** Molecular subtype-specific DEGSs unique to subtypes along with the regulation direction and |logFC| relative to other molecular subtypes, and those differentially regulated among different molecular subtypes relative to controls.

**Additional file 6. Table S6:** The clinical information of ASD patients in each molecular subtype, and the result of t test between each molecular subtype and the other two for each category of ADI-R scores.

## Data Availability

The single-nucleus RNA-seq data of ASD and controls can be downloaded from the website of https://autism.cells.ucsc.edu. Codes will be made available on request from the first author or the corresponding authors.

## Abbreviations

ASD: Autism spectrum disorder
DSM-5: Diagnostic and Statistical Manual for Mental Disorders, version 5
scRNA-seq: Single-cell RNA sequencing
snRNA-seq: Single-nucleus RNA sequencing
SNF: Similarity network fusion
AST-FB: Fibrous astrocytes
AST-PP: Protoplasmic astrocytes
IN-PV: Parvalbumin interneurons
IN-SST: Somatostatin interneurons
IN-SV2C: SV2C interneurons
IN-VIP: VIP interneurons
L2/3: Layer 2/3 excitatory neurons
L4: Layer 4 excitatory neurons
L5/6: Layer 5/6 corticofugal projection neurons
L5/6-CC: Layer 5/6 cortico-cortical projection neurons
Neu-mat: Maturing neurons
Neu-NRGN-I: NRGN-expressing neurons I
Neu-NRGN-II: NRGN-expressing neurons II
OPC: Oligodendrocyte precursor cells
GSVA: Gene set variation analysis
MSigDB: Molecular signatures database
DEG: Differentially expressed gene
DEGS: Differentially expressed gene set
LMM: Linear mixed model
LRT: Likelihood ratio test
logFC: Log2 fold change
SFARI: Simons Foundation Autism Research Initiative
ADI-R: Autism Diagnostic Interview-Revised
PLS: Partial least squares
